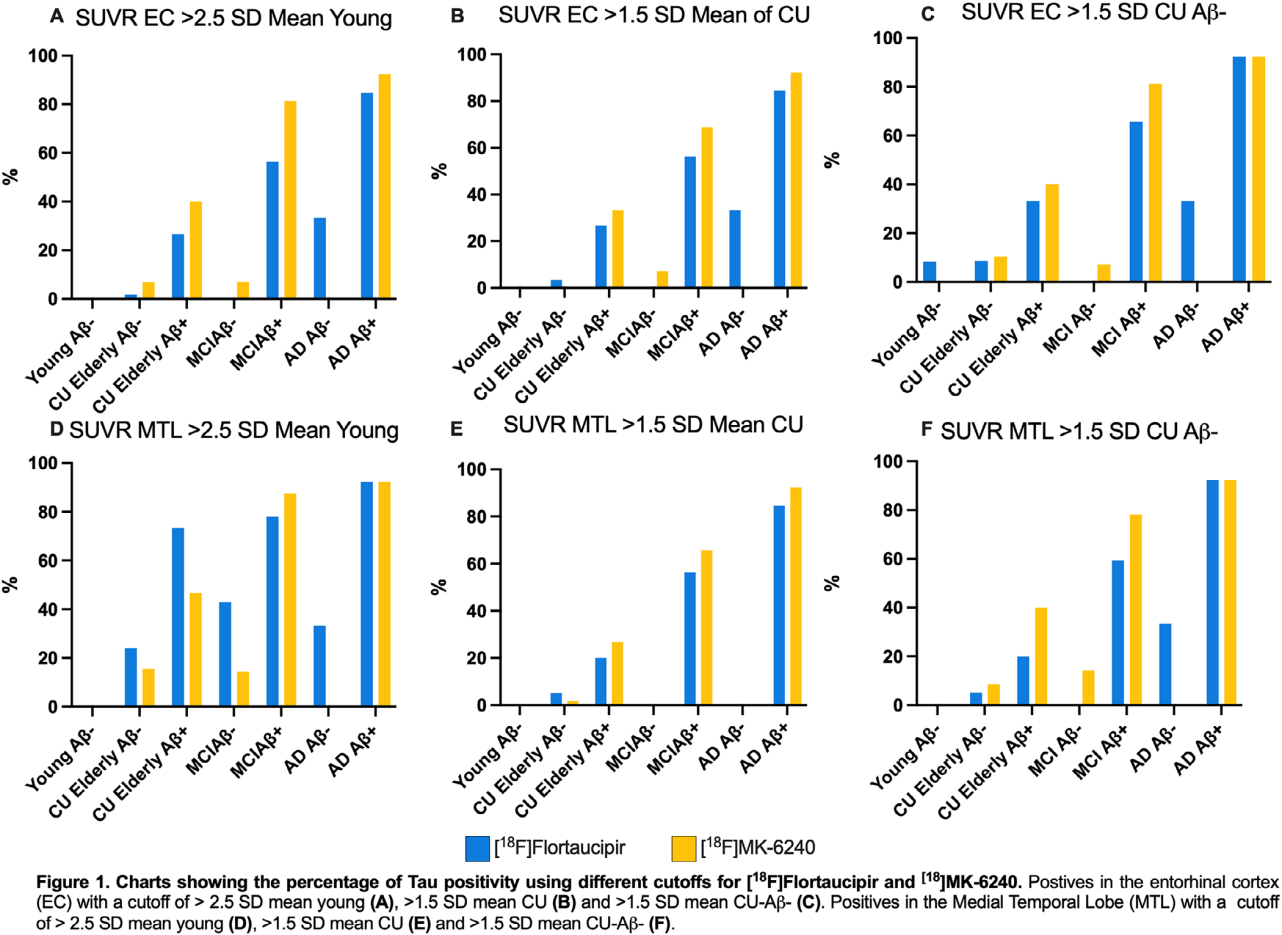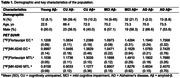# Impact of anchoring cutoff point of tau positivity on CU young or older adults using Flortaucipir and MK‐6240 – Head Study

**DOI:** 10.1002/alz.092793

**Published:** 2025-01-09

**Authors:** Emma Patrice Ruppert, Guilherme Povala, Guilherme Bauer‐Negrini, Firoza Z Lussier, Pamela C.L. Ferreira, Bruna Bellaver, Livia Amaral, Hussein Zalzale, Markley Oliveira, Pampa Saha, Matheus Scarpatto Rodrigues, Sarah Abbas, Carolina Soares, Cynthia Felix, Marina Scop Madeiros, Elisa de Paula, França Resende, Paulo Caramelli, William E Klunk, Dana Tudorascu, William J. Jagust, Belen Pascual, Brian A. Gordon, Val J. Lowe, Hwamee Oh, David N. soleimani‐meigooni, Pedro Rosa‐Neto, Suzanne L. Baker, Tharick Ali Pascoal

**Affiliations:** ^1^ University of Pittsburgh, Pittsburgh, PA USA; ^2^ Universidade Federal de Minas Gerais, Belo Horizonte, Minas Gerais Brazil; ^3^ University of Pittsburgh Alzheimer's Disease Research Center (ADRC), Pittsburgh, PA USA; ^4^ Helen Wills Neuroscience Institute, University of California, Berkeley, Berkeley, CA USA; ^5^ Houston Methodist Research Institute, Houston, TX USA; ^6^ Washington University in St. Louis School of Medicine, St. Louis, MO USA; ^7^ Department of Radiology, Mayo Clinic, Rochester, MN USA; ^8^ Brown University, Providence, RI USA; ^9^ Memory and Aging Center, Weill Institute for Neurosciences, University of California, San Francisco, San Francisco, CA USA; ^10^ Translational Neuroimaging Laboratory, The McGill University Research Centre for Studies in Aging, Montréal, QC Canada; ^11^ Lawrence Berkeley National Laboratory, Berkeley, CA USA

## Abstract

**Background:**

Tau PET provides continuous measurements of tau tangle pathology in the human brain. However, establishing cutoffs is crucial for selecting individuals for treatment in clinical trials or practice. In the absence of postmortem data, PET cutoffs must be established using statistical methods based on what is considered normal tracer uptake. In this study, we tested the impact of various methods to determine tau positivity using two different tau PET tracers in individuals scanned head‐to‐head.

**Methods:**

We studied 147 individuals from Head‐to‐Head Harmonization of Tau Tracers in Alzheimer's Disease (HEAD) study with tau tangle PET scans with [^18^F]Flortaucipir and [^18^F]MK‐6240, and amyloid‐β (Aβ) PET. Tau deposition was measured with the standardized uptake value ratio (SUVR) of each agent in the Medial Temporal Lobe (MTL) and the Entorhinal Cortex (EC). To determine Tau positivity three different methods were used: >2.5 standard deviations (SD) than the mean of the young, >1.5 SD mean of the cognitively unimpaired (CU) and >1.5 SD mean of CUAβ‐.

**Results:**

Demographic characteristics of the study population are reported in Table 1. Using the cutoff >2.5 SD mean of young, [^18^F]Flortaucipir was positive in 35 (23.8%) and 69 (46.9%) individuals in the EC and MTL, respectively. [^18^F]MK‐6240 was positive in 49 (33.3%) and 58 (39.5%) individuals in EC and in MTL. Using >1.5 SD mean of CUAβ‐, [^18^F]Flortaucipir was positive in 38 (25.9%) and 45 (30.6%) individuals in EC and MTL, while [^18^F]MK‐6240 was positive in 51 (34.7%) and 50 (34.0%) in EC and in MTL (Figure 1).

**Conclusions:**

Our findings indicate variations in tau positivity when employing different methods based on either the young or CUAβ‐. [^18^F]Flortaucipir exhibited a higher rate of positive results when the method based on young individuals was applied in the MTL. Conversely, [^18^F]MK‐6240 showed more consistent and generally higher positivity when other methods were used for cutoff determination and/or in the EC region. Further research with a larger sample size is required to gain a better understanding of the optimal cutoff determination methods for these tracers.